# Fast routine assessment of MGMT promoter methylation

**DOI:** 10.1093/noajnl/vdaa170

**Published:** 2020-12-05

**Authors:** Ivana Bratic Hench, Rosa Della Monica, Lorenzo Chiariotti, Michel Bihl, Markus Tolnay, Stephan Frank, Jürgen Hench

**Affiliations:** 1 Institute for Medical Genetics and Pathology, Division of Neuropathology, University Hospital Basel, Basel, Switzerland; 2 CEINGE Biotecnologie Avanzate, Naples, Italy; 3 Department of Molecular Medicine and Medical Biotechnology, Università degli studi di Napoli “Federico II,” Naples, Italy

**Despite recent advances in targeted diffuse glioma therapy, temozolomide still remains a therapeutic mainstay that rests on MGMT promoter methylation state as key predictor. Despite its importance in clinical decision making, MGMT testing has neither been standardized nor is it rapidly available. We, therefore, implemented an integrative diagnostic pipeline, comprising a methylation-specific PCR (MSP) complemented by a STP-27-based MGMT promoter assessment^1^ within methylation array analysis. Here, we report on the diagnostic precision of this approach that provides both a rapid neuro-oncology tumor board decision tool and a safe fallback method at affordable cost.**

Anonymized diagnostic and quality control data from 113 brain tumor patients from two centers (Basel, Switzerland; Naples, Italy) were analyzed. For each tumor, an integrated morphomolecular diagnosis (2016 WHO classification^2^), methylation array data, and the MSP result were available.

DNA from natively frozen (FF), formalin-fixed (FO), or formalin-fixed/paraffin-embedded (FFPE) biopsies was extracted using Maxwell FFPE DNA Purification (Promega) or Qiagen FFPE Tissue (Qiagen) kits. DNA was quantified photometrically (NanoDrop, Thermo Fisher) for methylation array and by a Qubit Fluorometer 2.0 (Thermo Fisher) for MSP. Two hundred to 400 ng of DNA was modified with sodium bisulfite (EZ Methylation DNA kit, ZymoResearch, cat.# D5001) according to the manual with exception of the last elution step (15 µL).

MSP primers targeting the DMR2 region of the MGMT promoter^3^ were used to amplify methylated and unmethylated sequences, yielding DNA products of 83bp (unmethylated) and 91bp (methylated). Twenty-five nanograms of DNA was amplified with HotStart Taq polymerase (Qiagen; cycler: 95°C/15 min; 35 cycles [95°C/45 s, 53°C/40 s, 72°C/1 min]; 72°C/10 min), visualized by gel electrophoresis (3% agarose gel, intercalating dye). Methylated MSP products only or both methylated and unmethylated bands were scored as “methylated”; unmethylated band only as “unmethylated” and samples negative for both reactions as “failed.” Controls: Meningioma samples (MGMT unmethylated) and universal methylated human DNA (ZymoResearch, cat.# D5011).

Methylation arrays (EZ Methylation DNA kit, ZymoResearch/Infinium EPIC, 850K, Illumina) were performed by a service provider (Life&Brain) according to the manufacturer’s protocols.

Classification, tools, and integrated diagnoses: Methylation classes, copy number profiles, and MGMT promoter methylation were determined from array data as reported.^1,4^ Unclear cases were verified by dimension reduction (http://www.epidip.org). Results were interpreted by board-certified neuropathologists in correlation with histology.

Methylation and coarse copy number data do not contain information traceable to individuals. According to local ethics and legal boards, such data are not classified as “personal health-related data” and are exempt from ethical permits for scientific evaluation.

**Figure 1.**
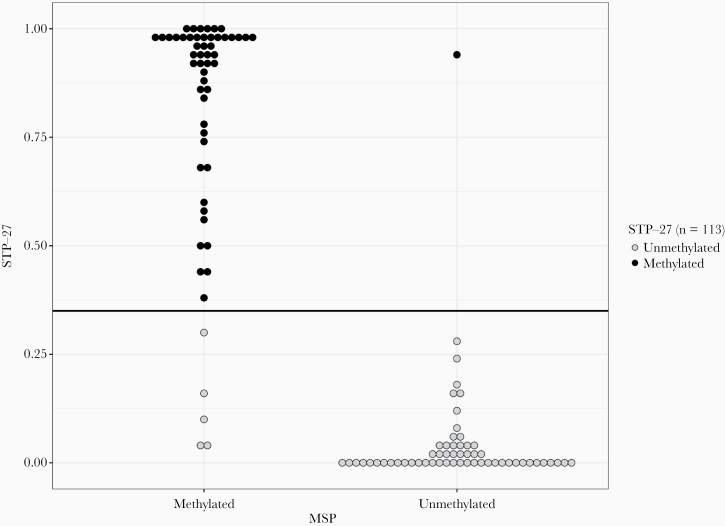
Bee swarm plot of STP-27 values, grouped by MSP result. Black line indicates STP-27’s decision cut-off.

Methylation-specific PCR-based (MSP) and STP-27 (tumor methylome-based)^1^ approaches to assess MGMT promoter methylation resulted in identical interpretation in 107/113 of cases (94.7%), with comparable concordance levels in both laboratories (*n* = 55/59; 93.2% [Naples], *n* = 52/54; 96.3%, [Basel]). Relative to STP-27, MSP produced false-positive (5/113) and false-negative (1/113) results (Figure 1). With the exception of IDH-mutant gliomas, MGMT promoter methylation status did not correlate with other tested major glioma subtypes (ie, GBM-RTKII, GBM-mesenchymal, GBM-RTKI; not shown). Impact of tumor cell content was negligible (not shown).

We present an easy-to-implement approach to fast and precise brain tumor diagnostics. Whereas nanopore sequencing-based tumor methylation profiling (nanoDx),^5^ implementable in most laboratories in developed countries, can drastically minimize temporal gaps between intraoperative consultations and diagnostic methylation classification, separate MGMT testing—not yet achievable by selective nanopore sequencing^6^—remains necessary. The application of MSP for MGMT testing in conjunction with the nanoDx pipeline now enables high-precision molecular brain tumor diagnostics as well as neuro-oncological decision within 48 h.
